# Awake Glioma Surgery with Intraoperative Mapping: Predictors of Language Outcome and Survival

**DOI:** 10.3390/diagnostics16131964

**Published:** 2026-06-24

**Authors:** Klemen Krašovec, Mihela Petovar, Tilen Žele, Ninna Kozorog, Tomaž Šmigoc, Janez Ravnik, Blaž Koritnik, Tomaž Velnar

**Affiliations:** 1 Department of Neurosurgery, University Medical Centre Ljubljana, 1000 Ljubljana, Slovenia; tilen.zele@kclj.si (T.Ž.); tvelnar@hotmail.com (T.V.); 2 Clinical Department of Anesthesiology and Intensive Therapy, University Medical Centre Ljubljana, 1000 Ljubljana, Slovenia; mihaela.petovar@kclj.si; 3 Department of Neurosurgery, University Medical Centre Maribor, 2000 Maribor, Sloveniatomaz.smigoc@ukc-mb.si (T.Š.); janez.ravnik@ukc-mb.si (J.R.); 4 Institute of Clinical Neurophysiology, Division of Neurology, University Medical Centre Ljubljana, 1000 Ljubljana, Slovenia; blaz.koritnik@kclj.si; 5 Alma Mater Europaea University, Slovenska Ulica 17, 2000 Maribor, Slovenia

**Keywords:** awake craniotomy, glioma, brain mapping, language, direct electrical stimulation, neuropsychological tests, survival, surgical procedures, operative

## Abstract

**Background:** Awake craniotomy with intraoperative mapping is the standard of care for gliomas located in language-eloquent regions, enabling maximal safe resection while preserving functional integrity. This study aimed to identify clinical and intraoperative predictors of postoperative language worsening and overall survival in patients undergoing awake surgery for malignant glioma. **Methods:** In this retrospective multicenter cohort study, 37 patients with malignant glioma in the dominant hemisphere underwent awake craniotomy with intraoperative mapping. Clinical, radiological, intraoperative, and postoperative variables were analyzed. Language outcome was classified as unchanged or worsened. Univariable and parsimonious multivariable logistic regression analyses were used to identify predictors of language worsening. Overall survival was assessed using univariable Cox regression. **Results:** Postoperative language worsening occurred in six patients (16.2%). Increasing age was associated with higher odds of postoperative language worsening in univariable logistic regression (OR 1.12 per year, 95% CI 1.02–1.23, *p* = 0.019). Due to the limited number of outcome events, multivariable logistic regression was not performed. In survival analysis, increasing age (HR 1.10, 95% CI 1.05–1.16, *p* < 0.001) and WHO grade 4 (HR 18.15, 95% CI 3.91–84.19, *p* < 0.001) were associated with shorter overall survival. No statistically significant association between extent of resection and overall survival was detected in this small cohort. **Conclusions:** Awake glioma surgery with intraoperative mapping was associated with favorable language outcomes in most patients at the 3-month follow-up. Increasing age was associated with postoperative language worsening in univariable analysis. These findings should be interpreted as exploratory because of the limited sample size and low number of outcome events. Larger prospective studies with standardized longitudinal language assessment are needed.

## 1. Introduction

Maximal safe resection of malignant glioma while preserving functional integrity represents the cornerstone of contemporary neurosurgical management. The evolution of intraoperative brain mapping has enabled reliable delineation of eloquent cortical and subcortical structures from tumor tissue, thereby facilitating maximal safe resection [1,2,3]. In current practices, intraoperative mapping is primarily directed toward preservation of motor and language function [4].

During awake craniotomy, patients perform language tasks tailored to tumor location while cortical and subcortical regions are interrogated using intraoperative neurophysiological monitoring. Direct cortical and subcortical stimulation permits functional identification of eloquent pathways and supports real-time surgical decision-making [5]. Advances in operative technique and improved understanding of functional neuroanatomy have expanded the extent of resection (EOR) achievable without compromising neurological function. Greater EOR has consistently been associated with prolonged overall survival (OS) and progression-free survival in patients with glioma [2,3,6].

Intraoperative mapping with direct electrical stimulation (DES) has been shown to improve functional and oncological outcomes. DES enables precise localization of cortical and subcortical language networks, whose injury may result in permanent postoperative deficits [2]. In most centers, intraoperative language assessment during DES relies on picture naming and counting tasks [7]. However, these paradigms provide only limited evaluation of higher-order language processing and do not adequately assess automatic speech, lexical retrieval, or complex expressive and receptive functions essential for effective communication.

In addition to intraoperative assessment, systematic preoperative and postoperative neuropsychological evaluation is strongly supported in the literature. Deficits in higher cognitive functions are frequently subtle, may escape detection during routine neurological examination, and are often underrecognized by patients and caregivers. Structured neuropsychological assessment is therefore recommended to establish baseline function, evaluate postoperative outcomes, monitor longitudinal changes, and guide cognitive rehabilitation strategies aimed at compensating persistent deficits [8].

In addition to intraoperative DES, modern surgical planning increasingly incorporates advanced preoperative mapping techniques. Diffusion tensor imaging tractography (DTI) enables visualization of critical subcortical language pathways, including language-related tracts, and may support surgical trajectory planning [9]. Navigated transcranial magnetic stimulation (nTMS) provides non-invasive functional localization of cortical language regions and may further assist risk stratification [10]. The integration of preoperative mapping and intraoperative neuromonitoring provides the highest odds of gross total resection, particularly in language-eloquent lesions [11].

Although nTMS is not routinely available at our institutions and was not used in the present cohort, both nTMS and DTI represent important adjuncts to contemporary glioma surgery. Nevertheless, intraoperative DES remains the reference standard for real-time functional mapping during awake surgery.

The primary aim of this study was to explore factors associated with postoperative language worsening in patients undergoing awake surgery in Slovenia. The secondary aim was to explore the relationship between EOR and OS.

## 2. Materials and Methods

This retrospective multicenter cohort study was conducted at two tertiary neurosurgical centers in Slovenia. Patients aged between 18 and 75 years with radiological findings consistent with malignant glioma located in the dominant hemisphere were included.

All patients underwent awake craniotomy with intraoperative language mapping using DES. Surgical procedures were performed according to institutional awake-mapping protocols. Neuronavigation was used in all cases. Fluorescence-guided surgery with 5-aminolevulinic acid (5-ALA) was routinely used in patients with suspected high-grade glioma according to institutional practice. DTI was used when available for preoperative planning and intraoperative orientation relative to eloquent subcortical pathways. Navigated transcranial magnetic stimulation was not routinely available during the study period and was therefore not used in this cohort.

Tumor localization was categorized according to functional–anatomical language regions obtained with magnetic resonance imaging (MRI). Language dominance was determined preoperatively using fMRI. The tumor–language relationship was classified as infiltration, displacement, contact, or no contact.

Preoperative neuropsychological and language assessment was performed during preoperative hospitalization. In patients receiving corticosteroids, assessment reflected the clinical language status at the time of preoperative evaluation. Systematic data on steroid-related language improvement before surgery were not available retrospectively. The neuropsychological preoperative battery included standardized evaluation of intellectual functioning, language, memory, visuo-constructive abilities, executive functions, and attention [12]. Preoperative speech function was graded on an ordinal scale (0 = normal, 1 = mild, 2 = moderate, 3 = severe disturbance). Postoperative language outcome was assessed at a 3-month follow-up and categorized as unchanged or worsened relative to preoperative baseline. Because the present study focused on the 3-month outcome, the term “postoperative language worsening” refers to language status at this specific time point. During awake mapping, intraoperative language assessment included counting, picture naming and spontaneous speech monitoring. In patients with pre-existing language deficits, tasks were adapted to individual baseline performance. Stimulation-induced disturbances were considered relevant when reproducible and clearly different from baseline performance.

Postoperative MRI was performed according to institutional protocol, typically within 24–48 h after surgery, to evaluate residual enhancing and non-enhancing tumor volume and determine EOR. Follow-up imaging was performed according to standard neuro-oncological practice. EOR was calculated based on established clinical guidelines using volumetric MRI and expressed as supramaximal resection (SMA), gross total resection (GTR), near total resection (NTR), subtotal resection (STR), partial resection (PR), or biopsy (B) [13].

Histopathological diagnosis was recorded from final pathology reports using the 2021 WHO classification [14].

Continuous variables were assessed for distribution and, due to non-normality and small sample size, were presented as medians. Group comparisons between patients with stable and those with worsening postoperative language functions were performed using the Mann–Whitney U test for continuous variables and Fisher’s exact test or chi-square test, as appropriate, for categorical variables. Univariable logistic regression analysis was performed to explore factors associated with postoperative language worsening. Due to the limited number of events, multivariable logistic regression was not performed to avoid model overfitting and unstable estimates. OS was defined as the time from surgery to death or last follow-up. Survival analysis was conducted using the Cox proportional hazards regression model. Univariable Cox regression was used to assess the association between individual variables and OS. Due to the limited number of events, multivariable Cox regression analysis was not performed to avoid model overfitting. All tests were two-sided, and a *p*-value < 0.05 was considered statistically significant. Statistical analyses were performed using IBM SPSS Statistics for Windows, Version 29.0 (IBM Corp., Armonk, NY, USA). 

The study was conducted in accordance with institutional and national ethical standards.

## 3. Results

A total of 37 patients were included. Postoperative language worsening occurred in 6 patients (16.2%), while 31 patients (83.8%) had unchanged language function. Baseline characteristics are presented in Table 1.

### 3.1. Group Comparisons

Patients with postoperative language worsening were significantly older than those with stable language function (median 63 vs. 43 years, *p* = 0.025). No significant differences were observed in operative duration (median 194 vs. 190 min, *p* = 0.984), sex (*p* = 1.000), tumor laterality (*p* = 0.567), language dominance (*p* = 1.000), preoperative speech deficit (*p* = 0.696), intraoperative DES-induced disturbances (*p* = 1.000), tumor–language relationship (*p* = 0.601), or EOR (*p* = 0.261). Detailed results are provided in Table 2.

### 3.2. Predictors of Postoperative Language Worsening

**Figure 1:**
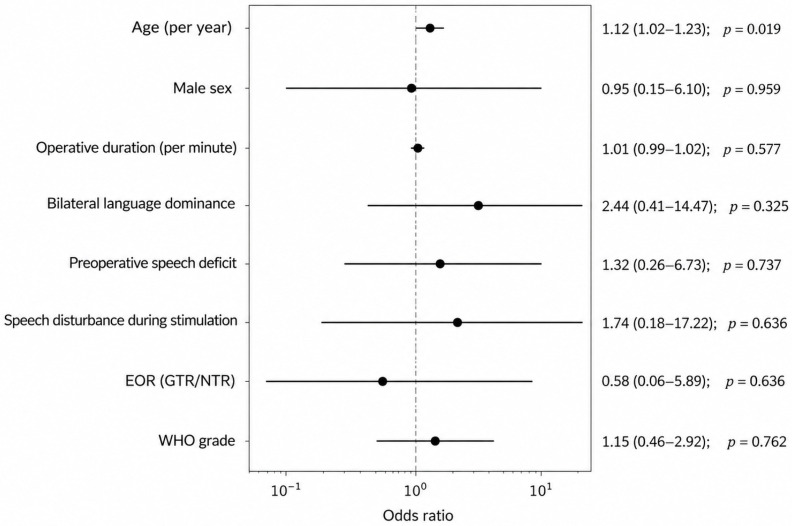
Forest plot of univariable logistic regression analysis for predictors of postoperative language worsening. Odds ratios with 95% confidence intervals are shown on a logarithmic scale. The dashed vertical line indicates no effect (OR = 1). Numeric estimates and *p*-values are shown to the right of each variable.

In univariable logistic regression, increasing age was associated with higher odds of postoperative language worsening (OR 1.12 per year, 95% CI 1.02–1.23, *p* = 0.019). No statistically significant associations were observed for the male sex (OR 0.95, 95% CI 0.15–6.10, *p* = 0.959), operative duration (OR 1.01 per minute, 95% CI 0.99–1.02, *p* = 0.577), bilateral language dominance (OR 2.44, 95% CI 0.41–14.47, *p* = 0.325), preoperative speech deficit (OR 1.32, 95% CI 0.26–6.73, *p* = 0.737), speech disturbance during DES (OR 1.74, 95% CI 0.18–17.22, *p* = 0.636), high EOR (GTR/NTR) (OR 0.58, 95% CI 0.06–5.69, *p* = 0.636), and WHO grade (OR 1.15, 95% CI 0.46–2.92, *p* = 0.762). Because only six postoperative language worsening events occurred, multivariable logistic regression was not performed. For better visualization, all estimates and their 95% confidence intervals are presented in Figure 1.

### 3.3. Survival Analysis

In univariable Cox regression analysis, increasing age was significantly associated with shorter OS (HR 1.10 per year, 95% CI 1.05–1.16, *p* < 0.001), as was WHO grade 4 (HR 18.15, 95% CI 3.91–84.19, *p* < 0.001) (Table 3). No statistically significant associations with OS were observed for sex (*p* = 0.969), tumor laterality (*p* = 0.758), language dominance (*p* = 0.618), preoperative speech deficit (*p* = 0.788), intraoperative DES-induced disturbance (*p* = 0.618), tumor–language relationship (*p* = 0.962) and EOR relative to PR (GTR *p* = 0.241, NTR *p* = 0.415, STR *p* = 0.213, Biopsy *p* = 0.998). However, the number of patients undergoing GTR or NTR was small, limiting statistical power to evaluate the prognostic role of EOR.

### 3.4. Key Findings

Postoperative language worsening occurred in 16.2% of patients. Increase was associated with worse functional outcome in univariable analysis. In survival analysis, increasing age and WHO grade 4 were significantly associated with shorter OS in univariable analysis. No other variables were significantly associated with functional or survival outcomes.

## 4. Discussion

In this multicenter retrospective cohort study, we explored factors associated with postoperative language outcomes and OS in patients undergoing awake glioma surgery with intraoperative mapping. The principal finding is that increasing age was associated with postoperative language worsening in univariable analysis. In survival analysis, increasing age and WHO grade 4 were associated with shorter OS. No statistically significant association between EOR and OS was detected; however, this finding must be interpreted cautiously because of the small cohort size and limited number of patients undergoing GTR or NTR.

### 4.1. Functional Outcomes and Language Preservation

The rate of postoperative language worsening in our cohort (16.2%) is consistent with previously reported outcomes following awake craniotomy for language-eloquent gliomas. Transient postoperative language worsening is common after awake craniotomy, occurring in approximately 53% of patients undergoing resection of language-eloquent high-grade gliomas, though permanent deficits (>3 months) occur in only about 9%, underscoring the effectiveness of intraoperative mapping strategies in preserving essential functional networks [15]. Preoperative language deficits, intraoperative anomia and production errors are a strong predictor of both acute and short-term postoperative language impairment [16]. However, this was not confirmed in our cohort.

A key and clinically relevant finding of this study is that older age was significantly associated with postoperative language worsening in univariable analysis. This observation aligns with prior longitudinal studies demonstrating that older patients are at higher risk of persistent aphasic deficits following awake surgery. In a prospective longitudinal study of 153 awake craniotomies with language mapping, age >40 years was identified as an independent risk factor for persistent aphasic disturbance at 7 months postoperatively (*p* < 0.02). While most patients experienced resolution of new postoperative language worsening, older patients showed reduced capacity for functional recovery [17]. Importantly, this association likely reflects age-related differences in neuroplasticity and functional reserve, rather than chronological age alone. Reduced capacity for cortical reorganization and diminished recruitment of alternative language networks in older individuals may limit recovery following surgical disruption of eloquent pathways or perilesional regions to restore language function [18,19].

In contrast to several previous reports, intraoperative DES-induced language disturbances were not predictive of postoperative deficits in our cohort. This finding contrasts with previous reports, where identification of eloquent areas through DES, while essential for surgical planning, paradoxically increased the risk of postoperative deficits compared to negative mapping (21% vs. 9% worsened deficits), likely reflecting proximity of the functional cortex to the tumor [20]. This underscores the need for more nuanced integration of intraoperative mapping data with preoperative functional status and individual patient factors.

### 4.2. Extent of Resection and Functional Balance

No association was observed between EOR and postoperative language outcome. Although this finding may be influenced by limited statistical power, it is consistent with the principle that maximal safe resection can be achieved without compromising functional integrity when guided by intraoperative mapping. A systematic review by Bu et al. found that awake craniotomy with electrical stimulation reduced late (≥3 months) language deficits and overall neurological deficits compared with general anesthesia, while simultaneously achieving better EOR [21]. For low-grade gliomas specifically, a 2025 meta-analysis found comparable EOR between awake and asleep surgery but significantly better long-term neurological outcomes with awake mapping [22]. This concept of “onco-functional balance” is central to modern glioma surgery and is supported by a growing body of evidence demonstrating that aggressive resection, when functionally guided, does not necessarily increase the risk of permanent deficits. At the same time, the absence of a detectable relationship between EOR and OS in our cohort contrasts with large-scale studies and meta-analyses that consistently demonstrate a survival benefit associated with greater resection. This discrepancy is likely attributable to the relatively small sample size and limited number of events, which reduce the ability to detect survival differences. Nevertheless, our findings reinforce the importance of interpreting oncological outcomes within the broader context of functional preservation, as postoperative neurological deficits may negate the survival benefits of more extensive resection. Importantly, the lack of a statistically significant association between EOR and OS in our cohort should not be interpreted as evidence against the established prognostic value of EOR. Rather, this result likely reflects limited statistical power, the small number of patients in the GTR and NTR categories, and the retrospective nature of the study.

### 4.3. Survival Outcomes and Language Worsening

In survival analysis, both age and WHO grade 4 were significantly associated with shorter OS in univariable analysis. WHO grade remains a well-established determinant of tumor aggressiveness and prognosis, while the impact of age likely reflects both biological factors and treatment tolerance. In contrast, postoperative language worsening was not significantly associated with OS. This finding should be interpreted cautiously given the limited number of events in the subgroup of patients with postoperative deterioration. Emerging evidence indicates that postoperative neurological impairment, particularly when persistent, may substantially impact survival, potentially offsetting the benefits of maximal resection. The lack of association observed in our study likely reflects the limited number of patients with postoperative deterioration, as well as the absence of longitudinal functional assessment distinguishing transient from permanent deficits. This highlights the need for time-sensitive evaluation of functional outcomes and their integration into survival analyses in future studies.

Information regarding adjuvant radiotherapy and chemotherapy was available for all patients. Treatment decisions followed contemporary international neuro-oncology guidelines and multidisciplinary tumor board recommendations and were largely determined by tumor grade. Patients with higher-grade tumors routinely received adjuvant radiotherapy and chemotherapy, whereas lower-grade tumors were managed according to standard clinical practice. As survival events occurred almost exclusively among patients with high-grade tumors who received standard adjuvant treatment, radiotherapy and chemotherapy were highly correlated with tumor grade and were therefore not included in the survival analyses.

The RANO Resect group review highlights that in glioblastoma, substantial neurological deficits completely negate the benefits of more extensive resection. In the GLIOMAP cohort, functional deterioration at 6 weeks (≥1 NIHSS point decline) was a negative predictor of outcome, with a statistical strength comparable to the positive effect of complete resection [23]. Aabedi et al. demonstrated that new postoperative neurological impairment was the key factor predicting survival across their entire cohort of IDH-wildtype glioblastoma patients—those with new impairments had a median survival of 11.6 months regardless of the extent of resection, versus 28.4 months in those without impairment who achieved complete CE resection [24]. The GLIOFO study introduced an onco-functional outcome (OFO) classification that powerfully illustrates this relationship: patients achieving GTR without functional loss (OFO 1) had a median survival of 21.0 months, compared to 12.0 months for those with GTR but functional loss (OFO 3), and 8.5 months for incomplete resection with functional loss (OFO 4) [25]. Awake craniotomy was significantly more likely to achieve OFO 1 (43% vs. 27%, OR 1.91) [25].

Notably, EOR was not significantly associated with survival in this cohort, which contrasts with prior studies demonstrating a survival benefit of more extensive resection. The GLIOMAP study demonstrated that awake craniotomy achieved significantly greater EOR and lower residual tumor volume compared with asleep resection, translating into a median OS advantage of 18.0 vs. 14.0 months and improved progression-free survival. Awake craniotomy was an independent predictor of GTR and OS in multivariable analysis [26]. A 2024 meta-analysis of 15 studies (2032 patients) confirmed that awake craniotomy yielded a mean EOR difference of +8.5%, with an OS benefit of approximately 2.9 months [27].

Meta-analyses consistently show that awake craniotomy with electrical stimulation is associated with approximately 30% reduction in mortality and a median survival advantage of approximately 4 months compared to asleep resection for high-grade gliomas in eloquent areas [28]. This benefit is driven by both greater EOR and fewer neurological deficits [27,29].

### 4.4. Implications for Language Mapping and Assessment Protocols

An important, yet underexplored, aspect of this study relates to the limitations of current intraoperative language assessment paradigms. In most centers, including those in our cohort, intraoperative mapping relies predominantly on picture naming and counting tasks. While these tasks are practical and widely used, they provide only a restricted evaluation of language function, primarily targeting lexical retrieval and speech production [30]. Such paradigms do not adequately capture higher-order language processes, including syntactic processing, semantic integration, and spontaneous speech, which are critical for effective communication in daily life. Consequently, patients classified as functionally “intact” based on intraoperative and early postoperative assessments may still experience subtle but clinically meaningful deficits [30,31].

Our findings, together with the absence of strong intraoperative predictors of outcome, support the need for more comprehensive, functionally relevant, and standardized language assessment protocols. This is particularly important in smaller linguistic populations, where validated and language-specific neuropsychological tools remain limited. The integration of structured preoperative, intraoperative, and longitudinal postoperative assessments may improve the sensitivity of outcome evaluation and better inform both surgical decision-making and rehabilitation strategies [21].

### 4.5. Limitations

This study has several limitations. The most important limitation is the small sample size and the very limited number of postoperative language worsening events. Consequently, multivariable logistic regression was not performed, and the results should be interpreted as exploratory. The study was not sufficiently powered to determine independent predictors of language outcome.

Although adjuvant treatment data were available for all patients, treatment allocation was strongly associated with tumor grade, following contemporary international neuro-oncology guidelines and multidisciplinary tumor board recommendations. Patients with higher-grade tumors routinely received adjuvant radiotherapy and chemotherapy, whereas lower-grade tumors were managed according to standard clinical practice. Because survival events occurred almost exclusively among patients with high-grade tumors who received standard adjuvant treatment, the independent effects of radiotherapy and chemotherapy on overall survival could not be meaningfully evaluated.

Furthermore, the retrospective design introduces a risk of selection bias. Tumor location was heterogeneous, and complete molecular data, including IDH mutation status and MGMT promoter methylation status, were not available for all patients. These missing variables represent important potential confounders in the survival analysis.

Additionally, TMS was not routinely available during the study period and was not used in this cohort, which may limit comparison with centers using multimodal preoperative language mapping protocols.

Postoperative language outcome was assessed 3 months after surgery. While this time point is clinically relevant and reduces the influence of immediate postoperative changes, the study did not include longitudinal language assessments beyond this period. Therefore, the evolution of language function over time and the distinction between delayed recovery and permanent deficits could not be fully evaluated.

Finally, language assessment was not fully standardized across all phases of care, which may have limited the detection of subtle deficits and reduced comparability across patients. The lack of consistent use of validated, language-specific neuropsychological instruments represents a key methodological limitation and highlights an important area for future development. For more comprehensive evaluation, validated structured neuropsychological batteries adapted to the Slovenian language, such as the Slovenian Word Retrieval Test (STIB) and the Slovenian Verbal Fluency Test (STEF), should be used [32,33]. For optimal and objective assessment, language testing should be individualized according to functional–anatomical tumor localization, baseline language function, and patient-specific characteristics. Ideally, the same standardized Slovenian-language battery should be applied consistently in the preoperative, intraoperative, and postoperative phases to ensure accurate assessment of language changes.

Future studies should incorporate standardized and validated Slovenian adaptations of comprehensive neuropsychological test batteries to enable more reliable and reproducible evaluation of language outcomes.

## 5. Conclusions

Advanced intraoperative mapping during awake glioma surgery enables safe resection of tumors located in language-eloquent regions while preserving functional integrity. Awake glioma surgery with intraoperative mapping was associated with favorable language outcomes at a 3-month follow-up in most patients with dominant-hemisphere malignant gliomas. Increasing age was associated with postoperative language worsening in univariable analysis. Age and WHO grade 4 were associated with shorter OS, whereas postoperative language worsening was not. Because of the small sample size and limited number of outcome events, these findings should be interpreted as exploratory.

Larger prospective studies with standardized preoperative, intraoperative, and longitudinal postoperative language assessment, as well as complete molecular data, are needed to validate these findings.

## Figures and Tables

**Table 1:** Baseline demographic, clinical, radiological and surgical characteristics of the study cohort (*n* = 37). Values are presented as median (interquartile range, IQR) or number (%). * EOR data were available for 32 patients. Percentages for extent of resection were calculated using patients with available EOR data as the denominator.

Variable	Value
Age, years, median (IQR)	44 (36–58)
Sex, *n* (%)	
Male	25 (67.6%)
Female	12 (32.4%)
Tumor laterality, *n* (%)	
Left	32 (86.5%)
Right	5 (13.5%)
Language dominance, *n* (%)	
Left	16 (43.2%)
Bilateral	12 (32.4%)
Not available	9 (24.3%)
DTI tractography, *n* (%)	35 (94.6%)
Tumor–language relationship, *n* (%)	
Contact	16 (43.2%)
No contact	11 (29.7%)
Infiltration	8 (21.6%)
Not available	2 (5.4%)
Preoperative speech deficit, *n* (%)	
Mild (grade 1)	28 (75.7%)
Moderate (grade 2)	8 (21.6%)
Severe (grade 3)	1 (2.7%)
DES-induced language disturbance, *n* (%)	
Yes	28 (75.7%)
No	9 (24.3%)
Extent of resection, *n* (%) *	
Supramaximal resection (SMA)	0 (0%)
Gross total resection (GTR)	4 (12.5%)
Near-total resection (NTR)	5 (15.6%)
Subtotal resection (STR)	11 (34.4%)
Partial resection (PR)	11 (34.4%)
Biopsy	1 (3.1%)
WHO grade, *n* (%)	2 (5.4%)
Grade 1	2 (5.4%)
Grade 2	13 (35.1%)
Grade 3	9 (24.3%)
Grade 4	13 (35.1%)
5-ALA administration, *n* (%)	37 (100%)
Adjuvant chemotherapy, *n* (%)	32 (86,5%)
Adjuvant radiotherapy, *n* (%)	32 (86,5%)
Postoperative language worsening, *n* (%)	6 (16.2%)
Deaths during follow-up, *n* (%)	13 (35.1%)

**Table 2:** Values are presented as median or number (percentage). Group comparisons were performed using the Mann–Whitney U test for continuous variables and Fisher’s exact test or chi-square test for categorical variables.

Variable	Stable (*n* = 31)	Worsening (*n* = 6)	*p*-Value
Age (years), median	43	63	0.025
Operative duration (min), median	190	194	0.984
Sex			1.000
Male	21 (67.7)	4 (66.7)	
Female	10 (32.3)	2 (33.3)	
Tumor laterality			0.567
Left	26 (83.9)	6 (100)	
Right	5 (16.1)	0 (0)	
Language dominance			1.000
Left	13 (41.9)	3 (50.0)	
Bilateral	9 (29.0)	3 (50.0)	
Preoperative speech deficit			0.696
Mild (grade 1)	24 (77.4)	4 (66.7)	
Moderate (grade 2)	6 (19.4)	2 (33.3)	
Severe (grade 3)	1 (3.2)	0 (0)	
DES-induced disturbance			1.000
Yes	23 (74.2)	5 (83.3)	
No	8 (25.8)	1 (16.7)	
Tumor–language relationship			0.601
Contact	12 (38.7)	4 (66.7)	
No contact	11 (35.5)	1 (16.7)	
Infiltration	6 (19.4)	1 (16.7)	
Not available	2 (6.4)	0 (0)	
EOR			0.261
PR	9 (29.0)	2 (33.3)	
STR	9 (29.0)	2 (33.3)	
NTR	4 (12.9)	1 (16.7)	
GTR	4 (12.9)	0 (0)	
SMA	0 (0)	0 (0)	
Biopsy	0 (0)	1 (16.7)	

**Table 3:** HR—hazard ratio; CI—confidence interval. Estimates marked as not estimable reflect categories with no observed events. NE = not estimable because of insufficient outcome events in the subgroup.

Variable	HR (95% CI)	*p*-Value
Age	1.10 (1.05–1.16)	<0.001
Sex	0.98 (0.30–3.17)	0.969
Tumor laterality	0.79 (0.17–3.59)	0.758
Language dominance	1.36 (0.41–4.49)	0.618
Preoperative speech deficit	1.15 (0.43–3.07)	0.788
DES-induced disturbance	0.74 (0.23–2.41)	0.618
Tumor–language relationship		
Contact vs. no contact	0.97 (0.31–3.03)	0.962
Infiltration vs. no contact	0.26 (0.03–2.16)	0.212
Not available vs. no contact	NE	-
EOR (reference = PR)		
GTR	2.69 (0.51–14.07)	0.241
NTR	0.41 (0.05–3.51)	0.415
STR	0.25 (0.03–2.19)	0.213
SMA	NE	-
Biopsy	NE	-
WHO grade 4	18.15 (3.91–84.19)	<0.001

## Data Availability

The data presented in this study are available upon request from the corresponding author.
